# Identification and characterization of two novel cathelicidins from the frog *Odorrana livida*

**DOI:** 10.24272/j.issn.2095-8137.2018.062

**Published:** 2018-07-31

**Authors:** Ruo-Han Qi, Yan Chen, Zhi-Lai Guo, Fen Zhang, Zheng Fang, Kai Huang, Hai-Ning Yu, Yi-Peng Wang

**Affiliations:** 1 College of Pharmaceutical Sciences, Soochow University, Suzhou Jiangsu 215123, China; 2 Department of Bioscience and Biotechnology, Dalian University of Technology, Dalian Liaoning 116023, China; 3 School of Biology & Basic Medical Sciences, Medical College, Soochow University, Suzhou Jiangsu 215123, China

**Keywords:** Antimicrobial peptides (AMPs), Cathelicidins, *Odorrana livida*, OL-CATHs, Antimicrobial activity, Anti-inflammatory activity

## Abstract

Antimicrobial peptides (AMPs) are a group of gene-encoded small peptides that play pivotal roles in the host immune system of multicellular organisms. Cathelicidins are an important family of AMPs that exclusively exist in vertebrates. Many cathelicidins have been identified from mammals, birds, reptiles and fish. To date, however, cathelicidins from amphibians are poorly understood. In the present study, two novel cathelicidins (OL-CATH1 and 2) were identified and studied from the odorous frog *Odorrana livida*. Firstly, the cDNAs encoding the OL-CATHs (780 and 735 bp in length, respectively) were successfully cloned from a lung cDNA library constructed for the frog. Multi-sequence alignment was carried out to analyze differences between the precursors of the OL-CATHs and other representative cathelicidins. Mature peptide sequences of OL-CATH1 and 2 were predicted (33 amino acid residues) and their secondary structures were determined (OL-CATH1 showed a random-coil conformation and OL-CATH2 demonstrated α-helical conformation). Furthermore, OL-CATH1 and 2 were chemically synthesized and their *in vitro* functions were determined. Antimicrobial and bacterial killing kinetic analyses indicated that OL-CATH2 demonstrated relatively moderate and rapid antimicrobial potency and exhibited strong anti-inflammatory activity. At very low concentrations (10 μg/mL), OL-CATH2 significantly inhibited the lipopolysaccharide (LPS)-induced transcription and production of pro-inflammatory cytokines TNF-α, IL-1β and IL-6 in mouse peritoneal macrophages. In contrast, OL-CATH1 did not exhibit any detectable antimicrobial or anti-inflammatory activities. Overall, identification of these OL-CATHs from *O. livida* enriches our understanding of the functions of cathelicidins in the amphibian immune system. The potent antimicrobial and anti-inflammatory activities of OL-CATH2 highlight its potential as a novel candidate in anti-infective drug development.

## INTRODUCTION

Antimicrobial peptides (AMPs) are small gene-encoded peptides that possess direct antimicrobial activities against diverse microorganisms (Boman, 1998). AMPs are evolutionarily ancient weapons found widely in living organisms, from prokaryotic bacteria to eukaryotic mammals (Radek & Gallo, 2007; Zasloff, 2002; Zhang, 2015). According to their structures, AMPs can be divided into many different families. Generally, most are rich in basic amino acids and are cationic in the physiological environment (Nguyen et al., 2011; Radek & Gallo, 2007). AMPs can exhibit direct antimicrobial activity against microorganisms, including bacteria, fungi, viruses, and even parasites (Nguyen et al., 2011), though these activities and spectra differ among different peptides. Unlike conventional antibiotics, most AMPs target the microbial membrane by electrostatic adsorption, subsequently resulting in cell rupture. As this action occurs rapidly, it is unlikely to induce resistance in microorganisms.

Cathelicidins are a family of important AMPs found exclusively in vertebrates. Since their first discovery (Gennaro et al., 1989), hundreds of cathelicidins have been identified from diverse vertebrates, including mammals (Zaiou & Gallo, 2002), birds (Feng et al., 2011; Wang et al., 2011; Xiao et al., 2006), reptiles (Chen et al., 2017; Wang et al., 2008; Wei et al., 2015; Zhao et al., 2018; Zhao et al., 2008), amphibians (Hao et al., 2012; Wei et al., 2013), and fish (Chang et al., 2005; Maier et al., 2008). Cathelicidin precursors possess a conserved structural organization, including a N-terminal signal peptide (30 residues), highly conserved cathelin domain (99–114 residues), and heterogenic C-terminal mature peptide (12–100 residues) (Zanetti et al., 2000). Upon stimulation, these precursors are proteolytically processed and the mature peptides are released (Zanetti, 2004). Cathelicidins are multifunctional AMPs, many of which possess potent and broad-spectrum antimicrobial activities against a wide range of microorganisms (Zanetti, 2004; Zanetti et al., 2000). Some also possess additional functions and are actively involved in host immune modulation and disease resistance, such as chemoattraction and activation of immune cells and promotion of angiogenesis and wound healing (Kahlenberg & Kaplan, 2013; Wong et al., 2013).

Cathelicidins from mammals, especially human cathelicidin LL-37, have been studied extensively. However, cathelicidins from amphibians remain poorly understood, with only 10 cathelicidins identified to date. In the present study, we reported on the identification and characterization of two novel amphibian cathelicidins, named OL-CATH1 and 2, from the odorous frog *Odorrana livida*. The cDNAs encoding OL-CATH1 and 2 were cloned, peptides were chemically synthesized, and their structures and functions were determined.

## MATERIALS AND METHODS

### Frog collection and tissue preparation

Two adult *O. livida* specimens (female, weight=150–200 g) were captured from Tongren, Guizhou Province, China (N27.94${}^{\circ }$, E108.62${}^{\circ }$). No specific permissions were required for the sampling location/activity, and the present study did not involve endangered or protected species. After collection, the frogs were euthanized, and the tissues were removed quickly and stored in liquid nitrogen for later use. All animal experimental protocols were approved by the Animal Care and Use Ethics Committee of Soochow University.

### cDNA library construction and screening of cDNA encoding cathelicidins

The lung tissue of *O. livida* was ground into powder in liquid nitrogen and total RNA was extracted using Trizol reagent (Life Technologies, CA, USA). A cDNA library of the frog lung tissue was constructed using an In-Fusion SMARTer^TM^ Directional cDNA Library Construction Kit (Clotech, Palo Alto, CA, USA). The experiment was conducted strictly according to the kit manual. The synthesized second-strand cDNA was used as the template for the following PCR screening.

According to the highly conserved cathelin domain sequence of previously characterized cathelicidins, an antisense degenerate primer (5′-WSCRCAGRYCTTCACCTCC-3′) was designed and coupled with a 5′ sense primer (5′-AAGCAGTGGTATCAACGCAGAGT-3′) supplied by the kit to amplify the 5′ fragments of the cathelicidin encoded cDNA. The PCR procedure was: 5 min of denaturation at 94 °C; 30 cycles: denaturation at 94 °C for 30 s, primer annealing at 57 °C for 30 s, and an extension at 72 °C for 1 min. The last cycle was followed by an extension step at 72 °C for 10 min. The PCR product was purified by gel electrophoresis and cloned into the pMD19-T vector (Takara, Japan) for sequencing.

According to the acquired sequences of the 5′ fragments, a sense primer (5′-ATGGAGATCTGGCAGTGTGTGATAT-3′) was designed and coupled with a 3′ antisense primer (5′-TACGCGACGCGATACGCGAAT-3′) supplied by the kit to amplify the full-length sequence of the cDNA encoding cathelicidins. The PCR procedure was: 5 min of denaturation at 94 °C; 30 cycles: denaturation at 94 °C for 30 s, primer annealing at 58 °C for 30 s, and an extension at 72 °C for 1 min. The last cycle was followed by an extension step at 72 °C for 10 min. The PCR product was finally cloned into the pMD19-T vector and sequenced.

### Multi-sequence alignment

The cathelicidins used for multi-sequence alignment were obtained from the protein database at the National Center for Biotechnology Information (NCBI, https://www.ncbi.nlm.nih.gov/protein/?term=cathelicidin). To ensure the result was as accurate as possible, representative cathelicidins from mammals, birds, reptiles and fish were selected. All cathelicidins from amphibians identified so far were also included. ClustalX (v2.1) and GeneDoc (v2.7.0) software were used for multi-sequence alignment.

### Bioinformatic analysis and structure prediction of OL-CATHs

The physical and chemical parameters of the OL-CATHs were analyzed using the ExPASy Bioinformatics Resource Portal (http://www.expasy.org/tools/). Secondary structures of the OL-CATHs were predicted by a novel online computational framework PEP-FOLD3.5 (http://bioserv.rpbs.univ-paris-diderot.fr/services/PEP-FOLD3/) (Lamiable et al., 2016). Secondary structure components of the OL-CATHs were calculated by an online SOPMA secondary structure prediction method (https://npsa-prabi.ibcp.fr/cgi-bin/npsa_automat.pl?page=npsa _sopma.html).

### Peptide synthesis

The OL-CATHs were synthesized by a peptide synthesizer (GL Biochem Shanghai Ltd., China). The crude peptides were purified by RP-HPLC and analyzed by MALDI-TOF MS to confirm purity higher than 95%.

### Antimicrobial assay

A standard two-fold broth microdilution method was used to determine the antimicrobial activity of OL-CATHs, as described previously (Chen et al., 2017; Wei et al., 2015). Briefly, microorganisms were incubated in Mueller-Hinton broth (MH broth) at 37 °C to exponential phase and diluted to 1×10^6^ colony forming units (CFUs)/mL. Two-fold dilutions of OL-CATHs (50 µL) were prepared with MH broth in 96-well microtiter plates and mixed with equal volumes of microorganism dilutions. The plates were slowly shaken (100 r/min) at 37 °C for 18 h and the minimum concentrations (MIC) at which no visible microbial growth occurred were recorded. The conventional antibiotic ampicillin was used as a positive control. The antimicrobial activity of the OL-CATHs against *Helicobacter pylori* was determined using CFU counting, as described previously (Makobongo et al., 2009), with minor modification. The *H. pylori* 26695 and 11637 strains were incubated with MH broth (Oxoid, UK) containing 10% fetal bovine serum (FBS) at 37 °C in microaerophilic conditions produced using a 2.5-L AnaeroJar atmosphere generation system (Oxoid, UK) and AnaeroPack-MicroAero (Mitsubishi Gas Chemical Company, Japan). After growth, the bacteria were diluted with fresh MH broth (containing 10% FBS) to 1×10^4^ CFU/mL. Serial concentrations of the OL-CATHs were added to the bacteria and the samples were coated on MH plates (containing 10% FBS). The plates were incubated at 37 °C in microaerophilic conditions for 3 d and the colonies on the plates were determined. The MIC values at which no bacterial colony growth occurred were recorded.

### Bacterial killing kinetics assay

The bacterial killing kinetics of OL-CATH2 against *H. pylori* 11637 were determined according to previously described methods (Chen et al., 2017), with minor modification. Briefly, *H. pylori* 11637 was incubated in MH broth (containing 10% FBS) at 37 °C in microaerophilic conditions and diluted to 1×10^6^ CFU/mL with fresh medium. OL-CATH2 was added to the bacterial suspension to a final concentration of 5×MIC, and the mixture was incubated at 37 °C in microaerophilic conditions for 0, 10, 20, 30, 45, 60, 90, 120 and 180 min. At each time point, aliquots (50 µL) were removed and diluted 1 000 times with fresh MH broth (containing 10% FBS). We coated 50 µL of each dilution on MH agar plates (containing 10% FBS), which were then incubated at 37 °C in microaerophilic conditions for 3 d, after which the number of viable colonies were counted. Ampicillin was used as the positive control and sterile deionized water was used as the negative control.

### Quantitative real-time PCR

The experiment was performed according to previous methods (Wei et al., 2015). Briefly, Brewer thioglycollate medium (Sigma-Aldrich, USA) was injected into the peritoneal cavity of C57BL/6 mice. The mice were euthanized 3 d later, and the mouse peritoneal macrophages (MPMs) were harvested. The MPMs were cultured in RPMI-1640 (containing 10% FBS, 100 U/mL penicillin and 100 µg/mL streptomycin, Gibco, USA) and plated in 96-well culture plates (1×10^4^ cells/well). After adhesion, the medium was replaced with fresh RPMI-1640 (containing 2% FBS, 100 U/mL penicillin, and 100 µg/mL streptomycin). The OL-CATHs (10 µg/mL) and lipopolysaccharide (LPS, 100 ng/mL, from *E. coli* 055:B5, Sigma-Aldrich, USA) were added to the wells and the cells were incubated for 6 h. At the end of incubation, the cells were collected for quantitative real-time PCR (qRT-PCR) to examine the gene expression levels of pro-inflammatory cytokines TNF-α, IL-1β and IL-6. Trizol reagent (Life Tech, USA) was used to extract total RNA. A PrimeScript First-Strand cDNA Synthesis Kit (Takara, Japan) was used to synthesize the first-strand cDNA. A SYBR Premix Ex Taq^TM^ II (Tli RNaseH Plus) two-step qRT-PCR kit (Takara, Japan) was used to perform the qRT-PCR experiment on an ABI Prism 7000 Real-Time PCR System (Applied Biosystems, Carlsbad, CA, USA). Cycle counts of the target genes were normalized to the GAPDH gene, and their fold changes were calculated. The primers used for qRT-PCR are listed in Table 1.

**Table 1:** Primers used for quantitative real-time PCR

Name	Forward (5${}^{\prime }$–3${}^{\prime }$)	Reverse (3${}^{\prime }$–5${}^{\prime }$)
TNF-α	CGGTGCCTATGTCTCAGCCT	GAGGGTCTGGGCCATAGAAC
IL-1β	ATGGCAACTGTTCCTGAACTC	GCCCATACTTTAGGAAGACA
IL-6	AGTTGCCTTCTTGGGACTGA	TCCACGATTTCCCAGAGAAC
GAPDH	GTGAAGGTCGGTGTGAACGGATT	GGAGATGATGACCCTTTTGGCTC

### Pro-inflammatory cytokine determination

The MPMs were cultured in RPMI-1640 (containing 10% FBS, 100 U/mL penicillin, and 100 µg/mL streptomycin, Gibco, USA) and plated in 96-well culture plates (1×10^4^ cells/well). After adhesion, the medium was replaced with fresh RPMI-1640 (containing 2% FBS, 100 U/mL penicillin, and 100 µg/mL streptomycin). The OL-CATHs (10 µg/mL) and LPS (100 ng/mL) were added to the wells and the plates were incubated at 37 ${}^{\circ }$C for 6 h, after which the cell culture supernatants were collected to determine TNF-α, IL-1β and IL-6 levels by enzyme-linked immunosorbent assay (ELISA, eBiosciences, USA).

## RESULTS

### Identification and characterization of OL-CATHs

**Figure 1:**
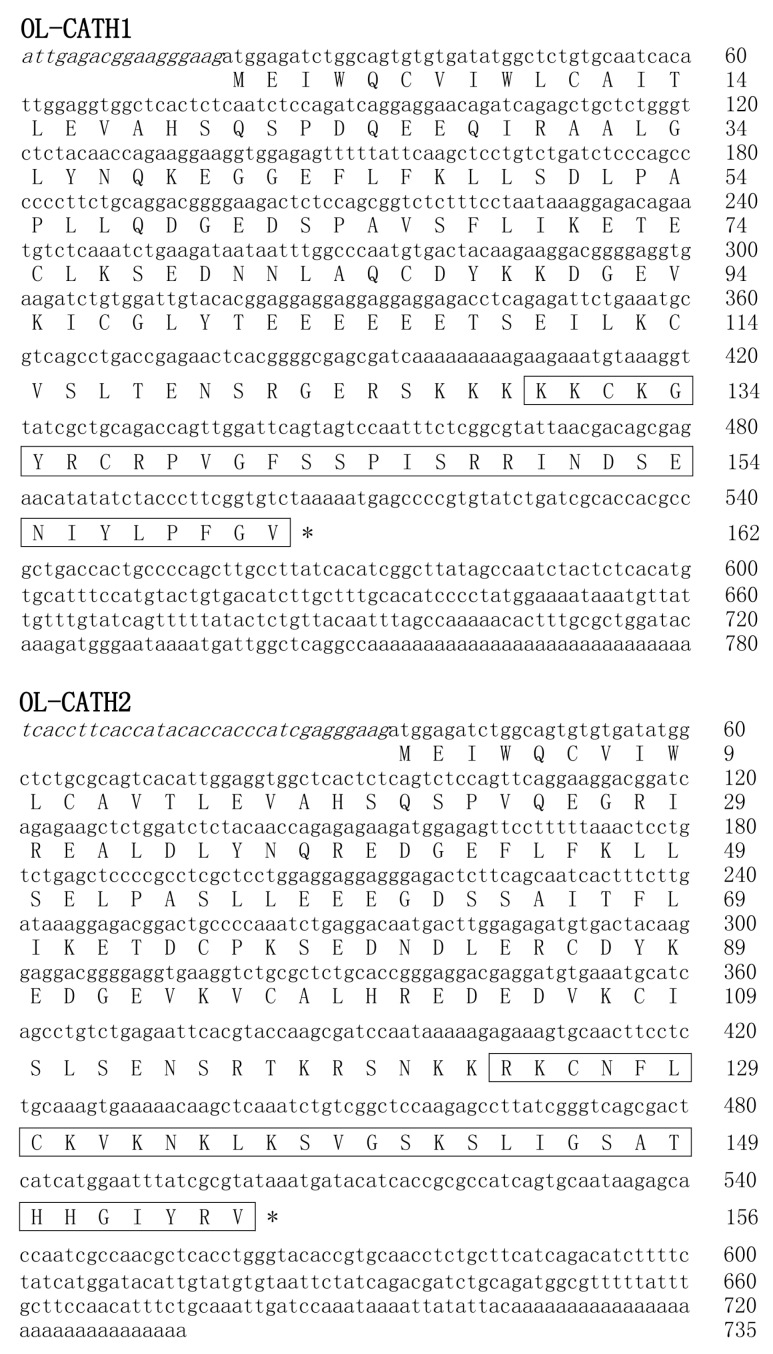
cDNA sequences encoding the OL-CATHs and predicted prepropeptide sequences

**Figure 2:**
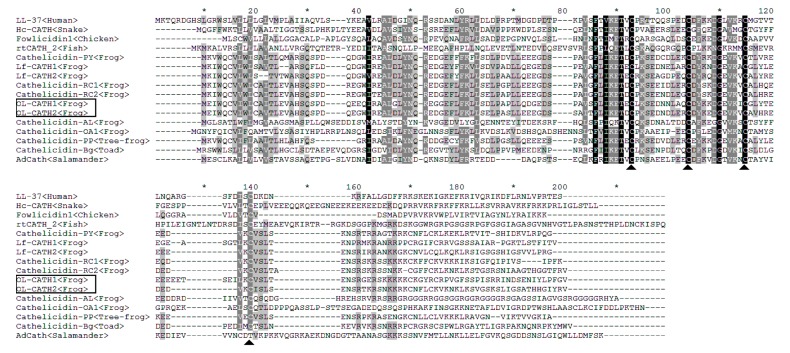
Multi-sequence alignment of the OL-CATHs with other representative cathelicidins

A *O. livida* lung cDNA library was successfully constructed using a cDNA library construction kit. From the library, two cDNA sequences encoding two novel cathelicidins were cloned (GenBank accession Nos.: MH282906–MH282907). The complete nucleotide sequences and translated amino acid sequences of the two cathelicidin precursors are shown in Figure 1. The two cDNAs encoding the OL-CATH1 and 2 precursors were 780 and 735 bp in length, respectively. The translated peptide precursors comprised 162 and 156 amino acid residues, respectively. Consistent with representative cathelicidins from other animals, the precursors of OL-CATH1 and 2 possessed an N-terminal signal peptide sequence, followed by a middle cathelin domain and a C-terminal mature peptide sequence (Figure 2). The signal peptide sequences and cathelin domains of the OL-CATHs shared high similarity with other cathelicidins. In particular, the four cysteine residues at the end of the cathelin domain were highly conserved within most of the cathelicidins, except for AdCath from salamander and Ss-CATH from salmon, in which the position of the fourth cysteine residue was different from that of other cathelicidins (Figure 2). Among the cathelicidins from frogs (including cathelicidin-PP from the tree-frog), there was a short acidic residue-rich fragment (D/E-rich) between the third and fourth cysteine residue of the cathelin domain. These frog cathelicidins also exhibited a high degree of similarity throughout the entire sequence (Figure 2).

5′-noncoding sequences are marked in italics. Putative mature peptides of OL-CATHs are boxed. Stop codon is indicated by an asterisk (*).

Identical residues are indicated in black and highly conserved residues are shaded. OL-CATHs are boxed and four highly conserved cysteines are indicated by a triangle (▲).

Previously, Wei et al. (2013) reported on the purification and characterization of a native cathelicidin (cathelicidin-PY) from the skin secretions of *Paa yunnanensis* and successfully cloned the cathelicidin from the skin cDNA library of the frog. The precursors of the OL-CATHs and cathelicidin-PY shared a highly conserved cleavage site for maturation (Figure 2), and accordingly the mature peptides of OL-CATH1 and 2 in the present study were predicted (Figure 1). OL-CATH1 was composed of 33 amino acid residues, including seven basic residues (three Lys, four Arg) and two acid residues (one Asp, one Glu), with an amino acid sequence of KKCKGYRCRPVGFSSPISRRINDSENIYLPFGV. OL-CATH2 was also comprised of 33 amino acid residues, including eight basic residues (six Lys and two Arg), with an amino acid sequence of RKCNFLCKVKNKLKSVGSKSLIGSATHHGIYRV. Consistent with cathelicidin-PY, the two cysteines in the sequences of OL-CATH1 and OL-CATH2 formed an intramolecular disulfide bridge. Sequence BLAST searching the NCBI protein database indicted that OL-CATH1 did not show significant sequence similarity with any known cathelicidins, whereas OL-CATH2 showed 61% similarity with cathelicidin-RC2 from *Rana catesbeiana*.

### Secondary structure modeling of OL-CATHs

The physical and chemical parameters of OL-CATH1 and 2 were analyzed by the ExPASy Bioinformatics Resource Portal (http://www.expasy.org/tools/) (Table 2). Both OL-CATH1 and 2 were found to be rich in basic amino acid residues (five and eight residues, respectively), with theoretical pI values of >9, implying that they are positively charged under physiological conditions.

**Table 2:** Physical and chemical parameters of OL-CATH1 and OL-CATH2

Peptide	Grand average of hydropathicity	Number of amino acids (*n*)	Net charge	Theoretical pI	Molecular weight
OL-CATH1	−0.582	33	5+	9.89	3 788.4
OL-CATH2	−0.291	33	8+	10.41	3 673.4

**Figure 3:**
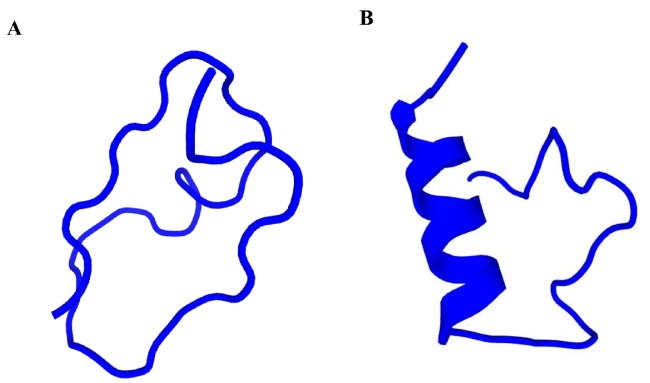
Secondary structure prediction of the OL-CATHs.

As shown in Figure 3, OL-CATH1 demonstrated a random coil secondary conformation, whereas the N-terminal of OL-CATH2 mainly adopted an α-helical conformation (Asn-4-Ser-15), as predicted by the online PEP-FOLD3 method. The predicted secondary structure components of the OL-CATHs using the SOPMA online prediction method indicated similar results. The predicted α-helix percentages for OL-CATH1 and 2 were 3.03% and 39.39%, respectively.

Structures of OL-CATHs were predicted by a novel online computational framework PEP-FOLD3.5 (http://bioserv.rpbs.univ-paris-diderot.fr/services/PEP-FOLD3/). A: OL-CATH1; B: OL-CATH2.

### Antimicrobial activity of OL-CATHs

The *in vitro* antimicrobial activities of the OL-CATHs were examined using a standard two-fold broth microdilution method. As shown in Table 3, OL-CATH1 did not show any detectable antimicrobial activity against the eight tested bacterial strains. In contrast, OL-CATH2 exhibited relatively moderate antimicrobial potency and was active against five of the eight tested bacterial strains, with MICs ranging from 9.38 to 75 µg/mL. In addition, OL-CATH2 appeared to be more potent against gram-negative bacteria than gram-positive bacteria and showed the most potent activity against the two *H. pylori* strains (MIC=9.38 µg/mL).

**Table 3:** Physical and chemical parameters of OL-CATH1 and OL-CATH2

Microorganisms	MIC (µg/mL)		
	OL-CATH1	OL-CATH2	Ampicillin
**Gram-negative bacteria**			
*Escherichia coli* ATCC25922	>200	37.5 (10.21 µmol/L)	4.69 (12.62 µmol/L)
*Helicobacter pylori* 26695	>200	9.38 (2.55 µmol/L)	18.75 (50.48 µmol/L)
*Helicobacter pylori* 11637	>200	9.38 (2.55 µmol/L)	9.38 (25.24 µmol/L)
*Pseudomonas aeruginosa* ATCC27853	>200	37.5 (10.21 µmol/L)	>200
**Gram-positive bacteria**			
*Staphylococcus aureus* ATCC25923	>200	75 (20.42 µmol/L)	9.38 (25.24 µmol/L)
*Bacillus subtilis* clinical strain	>200	>200	75 (201.9 µmol/L)
*Enterococcus faecalis* clinical strain	>200	>200	37.5 (100.9 µmol/L)
*Staphylococcus epidermidis clinical strain*	>200	>200	>200

MIC: Minimal inhibitory concentration. These concentrations represent mean values of three independent experiments performed in duplicates.

### Bacterial killing kinetics of OL-CATH2

According to the antimicrobial assay, OL-CATH2 showed the most potent activity against the two tested *H. pylori* strains. Therefore, we used *H. pylori* 11637 in the following bacterial killing kinetics assay. As illustrated in Table 4, at a concentration of 5×MIC, OL-CATH2 rapidly eradicated *H. pylori* 11637 cells within 45 min. In contrast, the positive control ampicillin needed at least 120 min to kill all bacterial cells. More importantly, the CFUs in the OL-CATH2-treated group remained unchanged at zero when the incubation time was extended to 180 min, implying that the effect of OL-CATH2 on bacteria was bactericidal rather than bacteriostatic.

**Table 4:** Killing kinetics of OL-CATH2 against *Helicobacter pylori* 11637

Time (min)	Colony forming units (×10${}^{3}$, CFUs/mL)								
	0	10	20	30	45	60	90	120	180
OL-CATH2	55	54.7	39	13.7	0	0	0	0	0
Ampicillin	58.3	56.3	70	64.3	60	39.3	16.7	0	0
Control	58.7	64	58.3	58.3	67.7	68.7	65.3	62.7	69.3

*Helicobacter pylori* 11637 was mixed with samples at concentration of 5×MIC for 0, 10, 20, 30, 45, 60, 90, 120 and 160 mins. The results represent mean values of three independent experiments performed in duplicates.

### Anti-inflammatory activity of OL-CATHs

**Figure 4:**
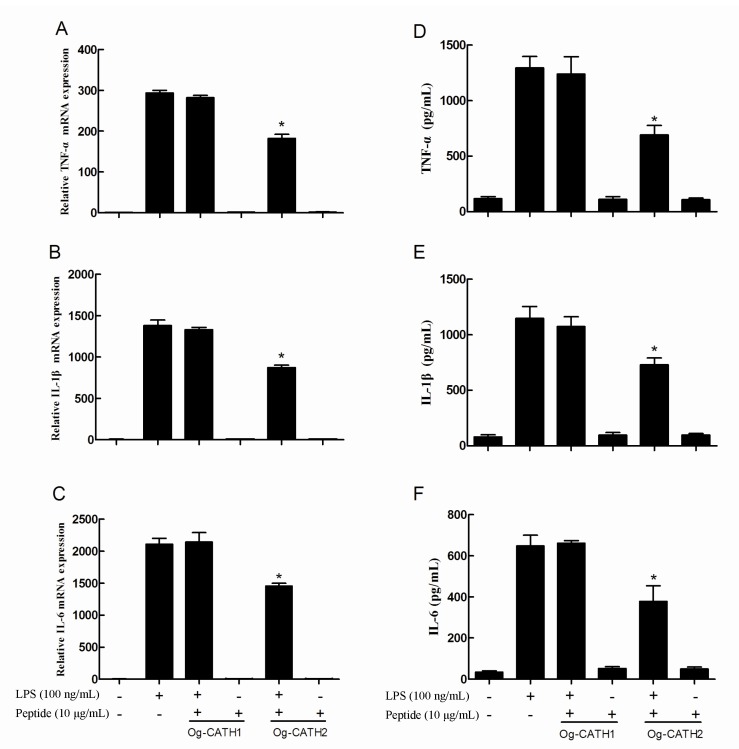
Anti-inflammatory activity of the OL-CATHs.

To study their anti-inflammatory activity, qRT-PCR was carried out to examine the effect of OL-CATHs on LPS-induced pro-inflammatory cytokine gene expression in MPM cells. As shown in Figure 4, compared with the untreated cells, 100 ng/mL of LPS significantly induced the transcription of TNF-α, IL-1β and IL-6 genes by 294-, 1 381-, and 2 110-fold, respectively. Furthermore, OL-CATH2 (10 µg/mL) significantly inhibited the LPS-induced gene expression of TNF-α (38%), IL-1β (37%) and IL-6 (31%), respectively. However, OL-CATH2 alone did not alter the transcriptional level of the pro-inflammatory cytokine genes. In addition, OL-CATH1 did not show any inhibitory effect on LPS-induced pro-inflammatory cytokine gene expression.

A: TNF-α mRNA. B: IL-1β mRNA. C: IL-6 mRNA. D: TNF-α production. E: IL-1β production. F: IL-6 production. Data are means±*SEM* of three separate experiments. *: *P*<0.05 significantly different to LPS-only group.

Furthermore, ELISA was carried out to study the inhibitory effect of OL-CATHs on LPS-induced pro-inflammatory cytokine production in MPMs. As shown in Figure 4, 100 ng/mL of LPS significantly induced the production of TNF-α, IL-1β and IL-6 to 1 294, 1 147, and 647 pg/mL, respectively. Consistent with the qRT-PCR results, OL-CATH2 significantly reduced the LPS-induced production of TNF-α, IL-1β and IL-6 to 691, 730 and 378 pg/mL, respectively. However, OL-CATH2 alone did not affect the production of the three pro-inflammatory cytokines. Furthermore, OL-CATH1 did not show significant inhibition of LPS-induced pro-inflammatory cytokine production. Overall, the above results indicate that only OL-CATH2 possessed potent anti-inflammatory activity.

## DISCUSSION

Amphibians are the first group of animals to bridge the evolutionary water-land gap. They are directly exposed to and interact with diverse ecological and physical factors, such as microorganisms, parasites, predators and environments (He et al., 2012; Xu & Lai, 2015). As the outermost organ, amphibian skin is directly exposed to harsh environments (Clarke, 1997), and is therefore endowed with an excellent chemical defense system comprised of many gene-encoded bioactive peptides (Clarke, 1997; He et al., 2012; Zhang et al., 2012). Over the past several decades, bioactive peptides from amphibian skin have been studied extensively (Li et al., 2007; Xu & Lai, 2015; Yang et al., 2009). In total, more than 100 families of peptides have been identified from diverse species of amphibians (Xu & Lai, 2015), with frogs belonging to the family Ranidae possessing the largest number of bioactive peptides in their skin. For instance, Li et al. (2007) identified 107 novel AMPs from an individual skin of *Odorrana grahami*. However, most previously detected AMPs are specifically expressed in the skin, and AMPs from other organs of frogs have not been studied extensively. Cathelicidins, an important AMP family expressed in many different organs of vertebrates, have also not been well studied in amphibians. To date, only 10 cathelicidins have been identified from amphibians, including cathelicidin-PY from the skin secretions of *Paa yunnanensis* (Wei et al., 2013), Lf-CATH1 and 2 from the spleen of *Limnonectes fragilis* (Yu et al., 2013), cathelicidin-RC1 and 2 from the lungs of *Rana catesbeiana* (Ling et al., 2014), cathelicidin-OA1 from skin secretions of *Odorrana andersonii* (Cao et al., 2018), cathelicidin-AL from the skin secretions of *Amolops loloensis* (Hao et al., 2012), cathelicidin-Bg from the ear-side glands of *Bufo gargarizans* (Gao et al., 2016; Sun et al., 2015), cathelicidin-PP from the skin secretions of *Polypedates puerensis* (Mu et al., 2017), and AdCath from the skin of *Andrias davidianus* (Yang et al., 2017).

In the present study, two novel cathelicidins (OL-CATH1 and 2) were identified from the lungs of the frog *O. livida*. The cloned cDNAs encoding OL-CATH1 and 2 were 780 and 735 bp in length, respectively (Figure 1) and the translated precursors were comprised of 162 and 156 amino acid residues, respectively. The precursors of the OL-CATHs possessed an identical structural organization to that of other representative cathelicidins, including an N-terminal signal peptide, highly conserved cathelin domain and C-terminal mature peptide (Figure 2). Of note, the position of the four cysteines at the C-terminus of the cathelin domain were highly conserved, which is a typical characteristic of the cathelicidin family of AMPs (Figure 2). The precursors of the OL-CATHs shared a highly conserved cleavage site for maturation with cathelicidin-PY, a novel cathelicidin isolated from the skin secretions of *Paa yunnanensis* (Figure 2), and accordingly the mature peptides of OL-CATH1 and 2 were determined (Figure 1). Protein BLAST searching indicted that OL-CATH1 did not show significant sequence similarity with any known cathelicidins, but OL-CATH2 showed 61% similarity with cathelicidin-RC2 from *Rana catesbeiana* (Ling et al., 2014).

Furthermore, the secondary structures of OL-CATHs were determined using bioinformatics prediction. According to the results, OL-CATH1 adopted a random coil secondary conformation, whereas the N-terminal of OL-CATH2 mainly adopted an α-helical conformation (Asn-4-Ser-15) (Figure 3). The α-helix percentages of OL-CATH1 and 2 calculated by the SOPMA online prediction method were 3.03% and 39.39%, respectively.

The OL-CATHs were chemically synthesized for further functional study. The *in vitro* antimicrobial assay results indicated that OL-CATH1 did not possess direct antimicrobial activity, whereas OL-CATH2 exhibited relatively moderate antimicrobial potency. Of note, OL-CATH2 showed strong activity against *H. pylori* bacteria (Table 3). As a result, *H. pylori* was selected for the further bacterial killing kinetics assay. Results demonstrated that OL-CATH2 exhibited efficient bactericidal effects against *H. pylori* cells within 45 min, which was more rapid than the effects of the positive control ampicillin (Table 4). The above results imply that OL-CATH2 plays an important role in host anti-infective responses by directly killing bacteria.

In addition to direct antimicrobial activity, some cathelicidins execute anti-infective functions in other ways, such as anti-inflammation, LPS neutralization, chemoattraction and activation of immune cells, and promotion of angiogenesis and wound healing (Chen et al., 2017; Kahlenberg & Kaplan, 2013; Steinstraesser et al., 2011; Wong et al., 2013). In the present study, we constructed an LPS-induced inflammatory cell model and studied the anti-inflammatory activities of the OL-CATHs. Consistent with the antimicrobial assay results, OL-CATH2 exhibited potent anti-inflammatory activity, though OL-CATH1 did not show any detectable effect. At a low concentration of 10 μg/mL, OL-CATH2 significantly inhibited the LPS-induced gene transcription and protein synthesis of several pro-inflammatory cytokines in MPMs (Figure 4). Thus, although OL-CATH2 could not kill the invading bacteria by direct antimicrobial activity at concentrations below the MIC, it still exhibited anti-infective functions by inhibiting excessive inflammation, which ultimately protected the host from pathogenic invasions.

In the present study, we reported on the identification and characterization of two novel cathelicidin family AMPs, i.e., OL-CATH1 and 2, from the odorous frog *O. livida*. Both peptides possessed conserved precursor construction but exhibited low sequence similarity of mature peptides with other known cathelicidins. OL-CATH2 exhibited moderate but rapid antimicrobial activity, and also possessed potent anti-inflammatory activity. Although the function of OL-CATH1 remains unclear, the identification of these OL-CATHs from *O. livida* has enriched our understanding of the functions of cathelicidins in the amphibian immune system. Furthermore, the potent antimicrobial and anti-inflammatory activities of OL-CATH2 make it a potential candidate in anti-infective drug development.
